# An Introduction to Botany

**Published:** 1837-07

**Authors:** 


### BRITISH AND FOREIGN MEDICAL REVIEW,

FOR JULY, 1837.

### PART FIRST.

### Analytical and Critical Reviews.

### Art. I.

1. *An Introduction to Botany.* By John Lindley, ph. d., f.r.s. l.s. and g.s., Professor of Botany in the University of London, and in the Royal Institution of Great Britain. *Second Edition.—London,* 1835. 8vo. pp. 580.

2. *Library of Useful Knowledge. Botany.—London,* 1835.—8vo. pp. 127.

3. *The Principles of Descriptive and Physiological Botany.* By the Rev. J. S. Henslow, m.a. f.l.s. &c., Professor of Botany in the University of Cambridge.—*London,* 1835. 12mo. pp. 322.

4. *Introduction à l'Etude de la Botanique, ou Traité Elémentaire de cette Science ; contenant l'Organographie, la Physiologie, la Méthodologie, la Géographie des Plantes, un aperçu des Fossiles Végétaux, de la Botanique Médicale, et de l'Histoire de la Botanique.* Par M. Alph. De Candolle, Professeur à l'Académie de Genève.—*Paris,* 1835. Tomes II. 8vo. pp. 1030.

*Introduction to the Study of Botany, or Elementary Treatise of that Science; containing Organography, Physiology, Methodology, and Botanical Geography ; with an Outline of Fossil Botany, of Medical Botany, and of the History of Botany.* By M. Alph. De Candolle, Professor at the Academy of Geneva.—*Paris,* 1835. Two Vols. 8vo. pp. 1030.

5. *Physiologie der Gewächse.* Von Ludolph Christian Treviranus, der Phil. und Med. Dr. und ord. Prof. an der Univ. zu Bonn, &c. Erster Band.—*Bonn,* 1835. 8vo. pp. 570.

*Vegetable Physiology.* By L. C. Treviranus, m.d. and ph. d., Professor in the University of Bonn, &c. Vol. I.—*Bonn,* 1835. 8vo. pp. 570.

6. *Nouveau Système de Physiologie Végétale et de Botanique, fondé sur les Méthodes d'Observation, qui ont été developpées dans le Nouveau Système de Chimie Organique; accompagné d'un Atlas de 60 Planches*

*d'Analyses*. Par F. V. Raspail.—*Paris*, 1837. Tomes II. 8vo. pp. 1257.

*New System of Vegetable Physiology and Botany, founded on the Modes of Observation which have been developed in the new System of Organic Chemistry; with an Atlas of* 60 *analytical Plates*. By F. V. Raspail.—*Paris*, 1837. Two Vols. 8vo. pp. 1257.

The appearance of five new treatises, within little more than twelve months, bearing on the study of Vegetable Physiology, besides a new and much improved edition of a former work, is an indication that the subject is now exciting a much more general attention than has hitherto been paid to it. Botanists have too frequently overlooked, in their eager pursuit of the systematic part of the science, the investigation of the structure and functions of the organs whose external form and arrangement afford them the means of classification. The study of the affinities of plants, however, upon which the Natural System is founded, requires an intimate acquaintance, not only with organography, or the internal as well as external conformation of their parts, but also with morphology, the department which treats of the laws regulating the arrangement and variation of organs, and which occupies in botany a station corresponding to that of philosophical anatomy in the study of the animal kingdom. The investigation of the elementary structure of organs naturally leads to the consideration of their functions; and hence arises the study of vegetable physiology, to prosecute which with success requires a correct and extensive knowledge of the two former departments, as well as some acquaintance with systematic botany.

Each of the treatises before us, with the exception of the last, is well fitted to impart much correct information on these subjects, which cannot but be interesting to the animal physiologist who takes an extended view of his science. The Introduction to Botany, by Dr. Lindley, is certainly the most valuable work of the kind in our language, incorporating a great amount of original observations with a judicious selection of the facts and opinions brought forwards by other authors. The department of Organography is very fully and ably treated of; but the division appropriated to Physiology appears to have been somewhat curtailed, many subjects of a highly interesting nature being left untouched. A considerable portion of the book is occupied with the explanation of terms used in systematic botany, and with rules for describing and naming plants, which we should like to see transferred to a separate volume, and their place in the present one occupied by an extension of the portion devoted to physiology, the arrangement of which, as it stands at present, we think capable of much improvement. It is scarcely possible, however, to praise too highly the mode in which the greatest part of the work is executed: the thorough acquaintance with his subject manifested by the author, and the facility with which he communicates his knowledge to others, render his explanations, even of the more abstruse points, easy to be comprehended; whilst the clearness and candour with which he displays the arguments on both sides of a disputed question incline us to accord, in almost all instances, with his opinion. The Treatise on Botany in the Library of Useful Knowledge, by the same author, is an excellent outline of vegetable anatomy and physiology, and we can strongly recommend it to such of our readers as are commencing the study of these sciences.

The work of Professor Henslow, being written for a popular series, is more adapted to the general than to the medical reader. It contains an extremely clear outline of many departments of the science, and forms an excellent introduction to the study of botany; but it is deficient in those higher and more general views which are so interesting to the advanced student. It cannot be doubted, from the author's high reputation as a botanist, that he could have introduced more of novelty in his illustrations; but any deficiency in this respect is counterbalanced by the lucid manner in which he has stated his views on several disputed questions of great interest, some of which we shall hereafter notice more particularly.

The Introduction of M. Alphonse De Candolle is principally an abridgment of his father's "Organographie" and "Physiologie Végétale," brought down to the date of its publication by the addition of new discoveries: it embraces a much more extensive range of subjects than either of the works already mentioned; and many parts of it may be read with great advantage by those who are already acquainted with them. Its first volume is devoted to anatomy and physiology, with the theory of classification; the second contains the characters of the natural families, with an excellent outline of botanical geography and of fossil botany, and a short but interesting history of botany.

The Treatise on Vegetable Physiology, by L. C. Treviranus, is the most comprehensive and elaborate, on this particular department, of any of the works before us. It contains not only his own opinions and researches, most of which have been already published in the Zeitschrift für Physiologie; but also a very complete abstract of the views of all eminent writers on each disputed point; and it will consequently be of great value to those who wish to enter fully into the investigation of the more obscure and intricate departments of vegetable physiology. We are glad to see so large a portion of the work devoted to the consideration of the Cryptogamia, which have been comparatively neglected by most English and French writers on Physiology. There are many valuable observations scattered through the works of those who have devoted themselves to the classification of this interesting division of the vegetable kingdom, which have not yet been properly incorporated; and, in proof of the degree in which investigations into the structure and functions of the Cellulares may assist us in the decision of several doubtful points in the physiology of vascular plants, we may refer to the beautiful discoveries made by Mirbel in his researches into the structure and mode of growth of the Marchantia Polymorpha.*

The volume of the Physiologie der Gewächse which has already appeared embraces only the functions of Nutrition; and, if those of Reproduction are treated of in the same elaborate manner, the work will be a valuable addition to those already existing. We certainly differ from the author in many of the opinions which he expresses; but the authorities on both sides are very fairly quoted, and much research is

* Nouv. Annales du Museum, tom. ii.

displayed in the abundance of references, which are of great value to the advanced student.

All the works which we have hitherto noticed profess to give an outline, more or less completely filled up, of the present state of the science of which they treat. That of M. Raspail has a far different aim; the object of the author being to demonstrate the fallacy of the usually received doctrines of vegetable anatomy and physiology, as well as of classification, and to prove that every observation hitherto made, which does not correspond with his own theories, has been falsified either through ignorance or design. M. Raspail was previously well known to us as possessing a remarkable degree of acuteness and ingenuity, as well as of originality of thought: his System of Organic Chemistry contains many observations of great value, mixed however with much that is erroneous, and which we hope to see retracted by the author in the forthcoming edition of his work. We applied ourselves to the perusal of the present volumes, therefore, with considerable expectations of the novelty and ingenuity of their contents; and in this respect we have certainly not been disappointed, since it appears to be the plan of the author to deny every fact, and to controvert every opinion, which is maintained by authors of established reputation, especially if they happen to belong to the Academy, his attacks on which are marked with a degree of acrimony and violence totally unworthy of the character of a man of science. In his eager pursuit of analogies and generalizations, the author has, we think, frequently allowed himself to be led astray from facts; and he has evidently, in many cases, resorted to observation with the view rather of confirming his preconceived theories than of employing them as the basis on which alone they should be constructed. In this mode of philosophising, he reminds us of the *savant* mentioned by Condillac, who thought that he had discovered a principle which was adequate to explain all the phenomena of chemistry; but, when he communicated his discovery to a skilful chemist, he was informed that the facts were unfortunately the reverse of what he had supposed. "Tell me what they are," was the philosopher's reply, "that I may explain them by my theory." The real or affected ignorance of the investigations of others, which is displayed by M. Raspail, is quite on a par with the supreme importance which he attaches to his own. Thus, in denying the existence of apertures in the Stomata, (which he regards simply as a modification of glands,) he takes no notice of the beautiful dissections of Ad. Brongniart, or of the remarkably complicated form in which Mirbel has shown that these organs exist in the Marchantia. Of neither could he be really ignorant, and his neglect of them appears to us to be readily explicable by the fact that his theory requires that the cuticle should be destitute of perforations.

Our criticism may appear harsh, but we can assure our readers that it is deserved. We are sorry to see that a man of M. Raspail's unquestioned ingenuity should be so far carried away by his zeal for novelty as to neglect that patient enquiry, as well as that freedom from prejudice, which are essential for the establishment of truth; and we do not consider it surprising that no one will give that implicit credit to his observations which he somewhat imperatively demands, but which is due only to men of higher reputation for accuracy. Were we to review all the passages which we have marked for special notice, our article would probably extend beyond the compass of M. Raspail's volumes; and we must therefore refer our readers to the work itself, in which they will find much to amuse as well as to interest, reserving a few of the fundamental propositions, however, for future consideration. The only quotation which we shall make is one which will afford a fair idea of the style and spirit which pervade the work; and we shall, contrary to our wont, give it in the original, as it would lose much of its point by translation.

"La rigueur mathématique, avec laquelle s'enchaînent les théorèmes de la démonstration de la deuxième partie, doit rappeler à la critique qu'elle ne doit rien écrire sans avoir médité au moins quelques jours: nous avons médité, nous, pendant douze années, et les jours de la plupart de ces années ont eu plus de vingt-quatre heures pour nous."

We should be glad to see the science of physiology based upon a more extensive generalization of the phenomena of vitality than has usually been thought necessary. The study of comparative anatomy is now recognized as the surest means of arriving at accurate results on many disputed questions; since the different forms of animals may be regarded, to use the language of Cuvier, as "so many kinds of experiments ready prepared by Nature, who adds to or deducts from each of them different parts, just as we might wish to do in our laboratories, showing us herself at the same time their various results." Vegetables present us with a greater simplification of the vital functions than is afforded by the lowest animal; since all the changes necessary to the support of the individual and the continuance of the species are performed without the influence or interference of those powers which are possessed, in a greater or less degree, by the whole animal kingdom. Hence, the physiologist may advantageously resort to the study of vegetable life for the explanation of many of the proximate causes of those phenomena which are complicated in the higher forms of organized beings by so great a variety of secondary influences. In many cases, too, he has the means of investigating in plants the changes produced by various agents on their structure and functions, with much greater facility than when studying the processes of the animal economy; and he has the power of more clearly distinguishing the effects of different vital stimuli.

The science of general physiology has, we regret to say, received little attention in this country, and we cannot point to any original work in our own language which professes to treat of it. The translation of the elaborate Comparative Physiology of Tiedemann has in part filled up the void; but this treatise is less fitted to impress on the mind of the student comprehensive views of the subjects which it discusses, than to present to those by whom they have already been acquired a store of facts which may be used in confirmation or illustration of them. In the hope of in some degree supplying to our readers the deficiency which we have ourselves felt, and with the desire of inducing a portion of them to follow up a course of investigation alike interesting and profitable, we shall devote the remainder of the present article to an outline of those departments of the structure and functions of vegetables, the knowledge of which is most important to the animal physiologist.

On the subject of the distinction between the two great kingdoms of organized nature, we fully partake of the opinion expressed by Professor Henslow, that *structure* presents us with no diagnostic mark by which we can separate the lower and most approximated groups of both from each other. "Perhaps," he remarks, "until the contrary shall have been proved, we may consider the addition of *sensibility* to the living principle as the characteristic property of animals; a quality by which the individual is rendered conscious of its existence or of its wants, and by which it is induced to satisfy those wants by some act of volition.' (*Principles*, p. 8.)

The power of locomotion has usually been regarded as the distinction between certain groups of aquatic animals and vegetables; and the difficulty of ascertaining whether such motions are voluntary or caused by accidental circumstances has led some of the German naturalists to describe beings as plants at one period of their existence, and animals at another. We cannot doubt that there is a definite boundary to each kingdom, although the imperfection of our means of observation will not always enable us to perceive it.

The *primary tissues* of vegetables offer an interesting object of study to the general anatomist; and the difference of opinion which still exists among botanists as to the precise nature of some of them, and their mode of formation, shows that there is still much room for investigation. Indeed, an examination into their nature demands not only considerable manual skill and dexterity in the use of the microscope, but, what is even more important, a perfect readiness to give up preconceived notions when they are inconsistent with observation, and a determination to consider nothing as proved until every mode of investigation has been employed with the same result. Every one who has examined doubtful objects by a high magnifying power must be aware how much is often left to the imagination of the observer; and it is not difficult to account for the great discrepancy which exists in the statements of animal as well as vegetable anatomists, all of whom, we have no doubt, conscientiously believed that they saw what they have described.

The views of Dr. Lindley on the subject of the *primary tissues* appear to us more definite and correct than those of any other writer, and we may therefore refer our readers to the statements contained in his Introduction to Botany, with which our own observations in general coincide. Those who feel an interest in general anatomy may also peruse with great advantage Mr. Slack's admirable paper on this subject,* which is accompanied with the best delineation of the elementary structures with which we are acquainted. From his researches, added to that of other observers, it appears that all the varied forms of vegetable tissue may be regarded as taking their origin from the simple cell; and this fact has an interesting connexion with the view now generally taken of the animal tissues, according to which they may be all reduced to the cellular, muscular, and nervous; all the parts which minister to organic life being essentially composed of the first of these and its numerous modifications. The different forms of vegetable tissue are usually classed under three general divisions, the *cellular*, *woody*, and *vascular*. The first of these is composed of adherent vesicles, more or less regular in form, usually

* Transactions of the Society of Arts, vol. xlix.

containing fluid, and sometimes having a spiral fibre generated in their interior. Woody tissue may be regarded as a very simple modification of the cellular, the vesicles being much elongated, and their sides possessing greater tenuity, combined with greater strength. The transitions from the one form to the other are almost imperceptible. *Vascular tissue* also consists of elongated cells, but these possess a spiral fibre in their interior, which appears destined to prevent the obliteration of the canal by pressure, when only filled with air; and in this respect it bears a remarkable analogy with the corresponding structure in the tracheæ of insects. We regard it as fully proved that the perfect *spiral vessels* are in some way connected with the respiratory system, being always found to contain air; but the various forms of *ducts,* produced by the partial rupture of the fibre, undoubtedly minister to the conveyance of fluid. It is probable that fluid may also be conveyed by *intercellular passages;* but we can by no means agree with M. De Candolle in the importance which he attaches to these canals in the economy of the plant, unless we are to regard the *vital circulation* of Schultz as taking place in them. We agree rather with Dr. Lindley in considering the *dotted ducts* as the principal means for the passage of the sap along the stem in most vegetables; and we generally accord with him, also, in respect to the cellular origin of these canals, though we think that some of Mr. Slack's observations render it probable that they may be occasionally modifications of spirally formed ducts.

With regard to the mode in which the vesicles of the different tissues are generated, little is certainly known. M. Raspail is of opinion that the membrane forming the inner lining of every vesicle is itself composed of other cellules in a rudimentary state, and that, when these are stimulated to development by a process analogous to that of impregnation, (the contact of male and female cellules,) new vesicles are formed within the old one, except when vessels are produced which are developed externally. Starting from this position, he gives a very plausible explanation of the formation of the trunk, leaves, flowers, and, in short, all the organs of the plant, each of which he regards as reducible to the type of a simple cell capable of generating others in its interior; and any cell possessed of this power is therefore supposed capable of producing any organ, if placed in circumstances fitting for its development. (§ 485.) We by no means wish to deny *in toto* the statements of M. Raspail; but we are confident that he is not arguing upon a sure foundation when he maintains that every new vesicle of cellular tissue is developed from the interior of a parent cell, like ovules in an ovary; since all the observations which have been made on the early development of the Cryptogamia concur in this, that new vesicles are added to the extremity of old ones, as any one may distinctly see in the Chara, and as Mirbel has demonstrated in his admirable memoir on the Marchantia. According to M. Raspail, every generating cell is composed of an external colourless envelope, and a second layer, composed of green globules, arranged in spirals which are the rudiments of the undeveloped cells. When the parent cell forms an entire organ by the development of these cells, and of their offspring, it is the external membrane which forms the general cuticle. M. Raspail has evidently generalized hastily on this subject from a few observations, and we cannot regard his doctrines as capable of universal application, although they may have some foundation in truth.

It is in the individual cells that the curious circulation of fluid takes place which was described a century ago by Corti, and to which the attention of botanists has been more recently directed by Amici. This phenomenon is best observed in the very elongated cells of Chara and Nitella, but it has also been witnessed in the transparent parts, especially the hairs, of many vascular plants, both terrestrial and aquatic; and it may be considered as probably existing in all cells at some period of their growth, being apparently connected with the functions of nutrition. It has therefore no analogy with the general circulation of animals; but we may derive an inference from it with regard to the possibility of the motion of fluid without any evident organ of impulsion.

It would not be difficult to show that many of the laws of organic development which have been suggested by the study of animal structures derive additional confirmation from their application to the vegetable kingdom. The botanist can point to the endless varieties of appearance and function assumed by the leaf as a striking illustration of the doctrine of unity of type; and the reduction of all the parts of the flower to the same elementary form by the rigorous application of the laws of morphology, affords a beautiful example of the principles of analogy. The higher forms of vegetables, like those of animals, pass, during their development, through a series of stages in which the structure of each organ bears considerable analogy with that which is permanent in some inferior beings. Thus, the embryonic vesicle, which is the rudiment of a vascular plant, apparently differs little in structure from the simple cell which constitutes the lowest form of cryptogamic vegetation; but the latter, as soon as it is formed, is capable of maintaining an independent existence, and of reproducing its species; whilst the former derives from nutriment previously assimilated by its parent the materials for the commencement of its development in the interior of the ovule, and cannot continue to grow by its own unassisted power until the rudiments of its complicated nutrient apparatus have been evolved. This *germinal vesicle* is filled with a whitish fluid, and is placed in the midst of the pulpy mass which constitutes the bulk of the ovule before impregnation. It seems at first to consist of nothing but mucus, but it gradually becomes firmer in texture, and absorbs from the tissue around it, increasing in size so as to fill up the whole or a large part of the ovule. Still, however, it possesses a supply of nutriment stored up in its own cotyledons, or in the albumen, which may properly be compared to the yolk-bag of the eggs of birds. This is its state at the period of the maturity of the seed, and thus it remains until excited by external stimuli to germinate, in which process a large quantity of carbonic acid is disengaged. Up to this time, it consists of cellular tissue and its simpler modifications; and, in structure, habits, and mode of existence, may be regarded as bearing a strong analogy with the lower cellular plants, especially the fungi, which, like it, deteriorate the air by the formation of carbonic acid, and, like it, also thrive best when well supplied with moisture and protected from light. As soon, however, as the plumula has elevated itself above the surface, and the cotyledons have acquired a green colour, the young plant begins to form woody tissue, but its vascular system is still imperfect. In this stage of its growth it may be regarded as corresponding with the mosses or Hepaticæ; and the presence of stomata on its cuticle would remind us of a similar structure in the curious Marchantia polymorpha. In a short time the true leaves are evolved, which repeat in a much more perfect manner the functions previously performed by the cotyledons; and the plant, having now exhausted the store of nutriment provided for it, is totally dependent upon itself for support. The stem now becomes provided with a regular fibro-vascular system, and the plant may be considered as having arrived at the level of the ferns: we must wait for full maturity before the reproductive organs are evolved, the appearance of which marks the highest type of vegetable organization.

Having thus attempted to point out a few of the transitory resemblances exhibited by the more highly organized plants to the permanent states of the lower, it would be interesting, did our space permit, to attempt to apply to the vegetable kingdom Mr. Macleay's ingenious principle, that in the lowest and most imperfect groups are sketched out the forms which are afterwards to be adopted in the higher divisions.* It would not be difficult to trace in the ever-varying forms of the Protophyta the outlines of the characters of the four remaining divisions of the vegetable kingdom; but on this subject we cannot at present dilate, and shall content ourselves with quoting an observation of that distinguished cryptogamist, M. Agardh: "Inter inferiores formas superiores sæpe efflorescunt, sed rudes et veluti experimenta; sic anticipationes formæ perfectioris in plantis inferioribus non raro obveniant; ut etiam in plantis superioribus regressus ad formam imperfectiorem."†

Amongst other general analogies between the animal and vegetable kingdoms, we must not overlook that of their chemical composition. Although carbon may be represented as the characteristic element of plants, as nitrogen of animals, it is now well known that the latter element occurs much more frequently in plants than was formerly supposed; and it is interesting to remark, that the vegetable principle into which it enters most largely is *fungin*, a substance confined to one of the groups which approaches nearest to animals in structure. We may also observe, that the mineral ingredients assisting in the formation of the organs of support in the lower animals are those which are usually found in plants. Thus, carbonate of lime, which is most frequent in vegetables, is most universally diffused through the hard parts of animals, constituting the whole of the skeletons of the massive Polypifera, the Mollusca, and forming a part of those of the Articulata and Vertebrata. In like manner, silex, which is the consolidating ingredient in the reticulated skeletons of the lower Porifera, the class of animals most allied to plants, also occurs, though sparingly, among the Algæ, and enters very abundantly into the structure of the higher plants, especially the Gramineæ. All the mineral deposits which we find in plants have, like those in the lower animals, a distinctly crystalline structure; and some of these substances are so insoluble as to be capable of being artificially crystallized by ordinary means. M. Becquerel, however, has succeeded in crystal-

* Horæ Entomologicæ, p. 223.

† Aphorismi Botanici, Part V. Lundæ, 1819.

lizing silex and the carbonate and oxalate of lime, by means of a long-continued and feeble current of galvanic electricity; and still more striking results of a like kind have been since obtained by Mr. Crosse,* which, we doubt not, were original on his part, but which had been anticipated, in great measure at least, by the experiments of the former gentlemen. It is not, perhaps, unreasonable to suppose that it is by similar means that the process is effected in plants, since a considerable quantity of electricity must be generated by the rapid evaporation of the water which holds these substances in solution. It might be argued from analogy that the calcareous granules in the organized skeletons of Vertebrata partake of the same crystalline arrangement, when first deposited, but that their form is afterwards altered by the constant absorption and renewal which takes place in true osseous textures.

The last general analogy we shall notice is the tendency to a spiral arrangement manifested in both kingdoms. All the higher plants may be regarded as constructed upon this plan, which is exhibited both in the attachment of the foliaceous appendages and in the formation of the stem itself: in both cases it might escape the notice of a superficial observer, but the universality of the fact is pointed out by De Candolle,† and has been more recently demonstrated by Braun, whose researches are detailed in the works at the head of the present article. Many stems have a tendency to twist on their own axes; and, when a lateral direction is given at the same time, the helical mode of growth is produced, so common in climbing plants. The stalk generally coils from right to left, but some species, like the hop, turn in a contrary direction; and in both cases the plant languishes and dies, if its natural mode of growth is interfered with. The complication of the forms of animals prevents our recognizing a similar arrangement in their higher groups; but it is the prevailing type of formation through a large portion of the Mollusca, and we believe that it has also been shown to govern the addition of new plates in the external skeleton of the Echinoderma. In the Mollusca, the usual direction of the spire is from right to left, but many genera invariably turn in the contrary way, and their shells are then said to be reversed. In some species the reversion is occasional, and it is not unfrequently met with in bivalve as well as spiral shells. There can be little doubt that, whatever cause produces the reversion of shells also effects the transposition of organs in the higher animals; and it would not seem improbable that the abnormal formation is in some way connected with the position of the embryo during its development, since we constantly find it existing on one side of double-bodied fœtuses.

Although, however, we regard the spiral mode of development as that which predominates in the vegetable kingdom, we can by no means assent to the speculations of M. Raspail, whose spiro-vesicular theory appears to us one of the most ingenious absurdities which we have ever encountered. The importance, however, which he attaches to this favorite hypothesis requires that we should not thus summarily dismiss it, and we shall therefore allow our readers to judge of it for themselves, stating it as nearly in his own words as the nature of an abridgment will allow.

* Report of the Meeting of the British Association, August, 1836.

† Organographie Végétale, tom. i. p. 155.

"The solution of the problem I am about to enunciate," he says, "will account for the whole system of vegetation; and with the three elements of each vesicle we shall have the means of organizing, according to positive formulæ, every plant already known or hereafter to be discovered." (§ 715.) The following is the problem thus pompously introduced:—"The generating cellule being given, with its three essential elements, to find in one of these elements the immediate cause of the disposition and symmetry of organs in every species of plant," (§716;) and it is thus solved. The globules, of which he regards the parietes of the vesicle as formed, and which are the rudiments of future organs, will not become developed until they receive a certain stimulating or fertilizing influence, which is conveyed to them by the spiral fibres winding round the interior of the cell. This influence cannot be conveyed to them by a single fibre, however, (and the existence even of this in every cell, be it observed, is a pure assumption;) two spires, a male and female, revolving in opposite directions, are required for this purpose, and it is only at their points of intersection that the rudimental globules become developed; an effect which cannot be produced by any number of spires revolving in the same direction. If the original cell, therefore, becomes a trunk, and the secondary vesicles are leaves or buds, the latter will be arranged on the first in a manner corresponding with the crossing of the spires; and, where these are equal in number, and revolve with the same rapidity in each direction, the mode of disposition will be *alternate.* To produce the *opposite, spiral,* or *verticillate* arrangement, we are required to suppose a variation in the number of the spires or in their relative velocity of revolution, by which their points of intersection, and consequently the organs formed by the development of the globules, may be brought to assume almost any position with regard to each other, as is demonstrated by M. Raspail in a succession of theorems.

In conceding to this theory the merit of great ingenuity, we conceive that we give it all the credit which it deserves; as an explanation of the principles of vegetable structure, we reject it for many reasons, of which the two following are the principal:—1. The theory is not supported by *any* observations, except a single one upon a Conferva, in which we are inclined to think that the fertility of the author's imagination supplied the place of perfection in his instrument; and it is inconsistent with the statements of those who have observed with the clearest and most powerful microscopes, as well as with unbiassed minds; all of whom agree in stating that the spiral fibre, where it exists, is usually single, and that, when two or more spires occur, they run in the same direction. 2. It assumes that the arrangement of organs is constant in each species, and it is therefore inconsistent with the well-known fact that in many plants the position of the leaves is constantly varying; those which are usually opposite or alternate becoming verticillate, and *vice versâ.* We say nothing of the hypothesis of male and female spires, and of the impregnation of the subjacent globules by their contact, the probability of which we may leave to the judgment of our readers; and we shall detain them no longer upon a question of so little practical utility, proceeding rather to the more strictly physiological department of our subject.

The existence and preservation of all organized beings is so intimately connected with the changes which are constantly taking place in their composition, that our simplest ideas of vitality are derived from the observation of these processes, the organs by which they are performed, and the concurrent circumstances on which they are dependent. In all living beings, whether animal or vegetable, the appropriation of matter from without, its conversion into a nutritious fluid whose elements supply materials for the growth and reconstruction of the fabric, and the excretion of the particles unfit for these purposes, constitute the sum of the vital acts by which the existence of the individual is maintained, and if we make due allowance for the differences occasioned by the possession of the faculties of sensation and voluntary motion, we shall find, on a close examination, that there is really a much greater resemblance in the mode by which the processes of nutrition are performed in both kingdoms, than a superficial view would lead us to suppose.

Thus with regard to the ingestion of aliment, it has been frequently maintained as a typical difference between vegetables and animals, that the former absorb their nutriment from the whole or from special parts of their external surface, whilst the latter receive it into internal cavities where it undergoes a certain degree of preparation, before absorption can be properly said to commence. "The spontaneous motion essential to animals," says Cuvier, "requires peculiar modifications even in such of their organs as are essentially vegetative. Unprovided with roots to penetrate the soil, and constantly to absorb nutrition, it was necessary that they should be able to place within themselves a supply of aliment, and to carry its reservoir along with them. Hence is derived the first character of animals; their intestinal cavity, from which passing through the pores and vessels (which may be considered a kind of internal roots) the nutritive fluid penetrates every part of their system, and sustains the whole." This view, though generally correct, requires to be greatly modified when we come to apply it to particular cases; for not only are there animals in which the most minute investigation has failed to detect an approach to an internal cavity, but late researches into the character of particular organs with which some plants are furnished, render it very doubtful whether any definition of a stomach could be framed, in which they would not be included.

In the lowest tribes of cellular plants, which present no approach to a vascular structure, absorption seems to take place by the whole surface in nearly an equal degree; and there is no transmission of fluid from one part to another, each portion of the tissue imbibing from the surrounding medium the nutriment which it requires. It was in this manner that the greater part of the infusorial animalcules were formerly supposed to derive their support; but the researches of Müller, Ehrenberg, and others, have established that their complicated digestive apparatus entitles them to a place in the animal scale far above that which has been usually assigned to them. The gemmules of the porifera are however certainly nourished by external absorption merely; Dr. Grant states that "as long as they retain this form, and for some time after their development in a fixed condition, they present no perceptible canals or cavities of any kind in their body; nor do the polypiferous animals while they continue in the same free state of ciliated moving gemmules. As the development of the porifera proceeds, minute openings are observed to form on the surface, which extend gradually through the body, producing internal canals which terminate superficially in vents or fecal orifices."* These internal canals are bounded only by a more condensed portion of the general cellular substance of the body, and may be considered as a simple extension of the external surface. They are incessantly traversed by currents of water which are drawn in by the minute pores, and expelled by the fecal orifices, and there are no distant cæca or stomachs for receiving and retaining the aliment thus conveyed into the body; every part of its texture appearing to be nourished by direct imbibition from the fluid in its neighbourhood. In this very simple organization we cannot help tracing a strong analogy with the mode of nutrition in cellular plants. We have noticed that in the lowest orders of these, the absorption is nearly uniform through the whole surface; but as soon as any specialization of this function takes place, as we may partially notice in the lichens, and still more in the fungi, (whose reproductive system is completely separated from the nutrient,) it is manifested in the development of filamentous processes of which the whole length seems concerned in the active imbibition of fluid. Hence the chief difference between the porifera and cellular plants in their mode of receiving nutriment, is that in the latter, the extension of the absorbing surface takes place externally; whilst the alimentary canals of the former may be compared to the ramified roots of a plant turned inwards; a difference analogous to that of the bronchial and pulmonary organs of aquatic and air-breathing animals.

Passing on to the vascular plants, we find each elementary fibre of their roots to consist of a bundle of fibro-vascular tissue covered by a lax cellular integument, and terminating in a blunt succulent point, which has the power of absorbing fluid with great rapidity. The *spongiole,* as this point has been termed, is sometimes spoken of as a distinct organ; but it is nothing more than the growing point of the root, which, with a few exceptions, lengthens only by additions to its extremity. The soft lax tissue of the newly formed part causes it to possess in an eminent degree the power of absorption; but as the fibre continues to grow, and additional tissue is formed at the extremity, that which was formerly the spongiole becomes consolidated into the general structure of the root, and loses almost entirely its peculiar properties. The vessels contained in the elementary filaments are portions of the general vascular system of the root which is in direct connexion with that of the stem, serving to convey the absorbed fluid to the organs where it is to undergo its elaboration. The most distinct analogy to this structure in the animal kingdom is perhaps to be found in the higher forms of the echinoderma, such as the holothuria, where the alimentary matter is absorbed from the parietes of the internal cavity by vessels corresponding to the mesenteric veins, by which it is conveyed to the respiratory organs. The roots of plants have been considered by some authors as analogous to the *lacteal* system of animals; but the erroneous nature of this comparison is evident when we reflect that scarcely any rudiments of this system are found in the invertebrata, and that in those classes whose organs of circulation are most nearly allied to those of vegetables, absorption is performed by the general vascular apparatus.

It is obvious, from these particulars, that the alimentary system of the

* Grant's Outlines of Comparative Anatomy, p. 311.

lower forms of animals and vegetables differs extremely little; for in both is fluid absorbed by the general surface, or by a continuation of it specially modified for the purpose; in both are the nutrient materials brought into close contact with the parts they are to supply, without an intermediate system of vessels; and in both also the function appears to be continuously performed without the interference of volition. In the higher classes of both kingdoms the apparatus assumes a more perfect and distinct form; but even in them we see in the power of superficial absorption, which is probably possessed in some degree by all, manifest traces of the primitive community of function which characterizes the general surface of the lower orders. This is manifested in vegetables by the important share which the leaves take in their nutrition; for not only is it through these organs that the greatest part of the carbon is derived which enters into the composition of the tissues of the plant, but they also appear capable of supplying, to a certain extent, any deficiency which may occur in the quantity of moisture supplied by the roots. It is an axiom in vegetable physiology laid down by De Candolle, that "when a particular function cannot, according to a given system of structure, be sufficiently carried into effect by the organ which is ordinarily destined to it, it is performed, wholly or in part, by another." Thus, it is obvious, that when the roots are absent or imperfect, or are implanted into an arid or barren soil, any plant which flourishes in such circumstances must derive its chief support either from the leaves, as in the epiphytal orchideæ and the greatest number of aërial parasites, or from the general surface, when it performs the functions of the leaves, as in the cacti: and if we consider the manner in which plants faded by the intense action of light and heat are refreshed by the natural or artificial application of moisture, we cannot doubt that absorption takes place, in these instances also, by the general surface as well as by the roots.

In many plants we may observe concavities in various parts, fitted for the reception of the moisture caught from rain, or condensed from dew; and the mode in which this is applied to the nutrition of the plant is not always evident. Frequently the fluid thus collected is conveyed to the roots, by which it penetrates to the interior of the plant; but in many cases it is preserved in receptacles which are usually formed by a modification of the leaf, and which vary in the completeness of their structure from the simple hollow formed in the leaves of the tillænsia or dipsacus, o the extraordinary pitchers (*ascidia*) of the nepenthes or dischidia. The exact office performed by the pitchers in the vegetable economy is perhaps not yet fully established. In nepenthes and sarracenia there is some doubt how much of the fluid is the produce of secretion, and what proportion is derived from without. It has been stated that one of its purposes is to attract and destroy insects, whose decomposition may be a source of nutriment to the plant, but this is doubtful. The object of the pitchers of the dischidia rafflesiana is, however, much less equivocal, and their structure far more complicated than in the preceding instances. This curious plant grows by a long creeping stem which is bare of leaves until nearly its summit; and, in a dry tropical atmosphere, the buds at the top would have great difficulty in obtaining moisture through the stem, and a sufficient supply is therefore furnished them by the pitchers, which store up the fluid collected from the occasional rains. "The cavity of the bag," says Dr. Wallich,* "is narrow and always contains a dense tuft of radicles which are produced from the nearest part of the branch, or even from the stalk on which the bag is suspended, and which enter through the inlet by one or two common bundles. The bags generally contain a great quantity of small and harmless black ants, most of which find a watery grave in the turbid fluid which frequently half fills the cavity, and which seems to be entirely derived from without." The earth has been justly spoken of as the common stomach of plants, supplying them with nutriment ready to be taken up by their absorbent system, whilst animals may be said to carry their soil about with them; in this curious plant, the failure of its regular means of support has called forth the addition of an organ, which, like the stomach of animals, serves as a receptacle for the supplies it may occasionally obtain, and principally differs from it in not being filled by an act of volition on the part of its possessor.

The absorbent system of vegetables seems to correspond remarkably with that of animals in the non-existence of any visible pores, through which the passage of fluid could take place. A vegetable anatomist would have concluded from the structure of the roots of plants, that the absorbents of the intestinal canal do not commence by open mouths; and the most recent investigations seem to confirm this opinion by direct observation. It cannot be doubted, however, that the pores of the spongioles must have a sensible diameter, by which their power of absorption is limited. If the roots of a plant be placed in coloured solutions, they take up only the most finely divided particles; and if placed in dilute saccharine or mucilaginous solutions, the watery part of the fluid will find its way through the spongioles and become available for the sustenance of the plant, while the greater part of the thicker material will be left behind. In like manner, when saline solutions of a certain strength are presented to the roots, the water only, with a small proportion of the salt is taken up; and the remaining part of the fluid is found to be more strongly impregnated with the salts than before. Such experiments, however, do not afford any evidence of that discriminating power in the spongioles which is so remarkable in the lacteals of animals, and would rather confirm the analogy which we have supposed them to bear with the general circulating system of the lower animals, the remains of the absorbing powers of which are perceived in the faculty now generally conceded to the veins of the vertebrata. It has, however, been recently demonstrated by Dr. Daubeny, that plants have to a certain extent the power of selection by their roots, since of three individuals of distinct species which were made the subject of experiment, no one appeared to have taken up the smallest quantity of nitrate of strontian, with a solution of which they were freely watered. (*See Lindley's Introduction*, pp. 308-9.)

Some interesting experiments by M. Payen† on the effects of a minute quantity of tannin in solution upon the absorbent power of the roots, seem to render it likely that the apparent rejection of particular substances which Dr. Daubeny has proved to exist, may depend upon some organic change produced by them in the delicate tissue of the spongioles.

* Plantæ Asiaticæ Rariores. Vol. II., p. 35.

† Annales des Sciences Naturelles. N. S. Botan. III. pp. 5-20.

It does not appear however that the selecting power is employed to prevent matter from being introduced into the tissue of the plant which is capable of exerting a deleterious influence upon it; for many substances are taken up by the roots which speedily put a stop to vital action if an opportunity is not afforded for their excretion. Further researches on this subject are much required; and we would suggest that they might be advantageously prosecuted in connexion with the investigation, (which can hardly be considered as yet concluded,) to what extent the function of the lacteals is confined to the absorption of chyle, and how far soluble foreign matters are taken up by the veins.*

The nature of the substances from which animals and vegetables respectively derive their sustenance has been held to constitute an essential difference between them; and, although some late writers have attempted to invalidate it by asserting that vegetables require organic matter for their support, and that some animals are nourished by inorganic substances, we shall endeavour to show that the distinction is generally if not universally applicable.

"There are, perhaps, only two forms of matter," says Dr. Lindley, "which can properly be called nutritive; the one is carbon, the other water. Soil in its natural state is filled with the remains of organic bodies, which decompose, and become converted into carbonic acid. In proportion to the abundance of these is soil fertile. If we look to the effects of manures, we shall find that in most cases, except when their object is to alter the soil mechanically, or to act as stimulants, as is probably the case with sulphate of iron, their energy is in proportion to their capability of forming carbonic acid. Yeast, for instance, which is one of the most active manures we have, is so from possessing, beyond all other substances, the power of exciting fermentation, and thus of causing the formation of carbonic acid among the vegetable matter which lies buried in the soil." (Pp. 305-6.)

It has been fully proved by the experiments of Payen, that those soils which afford the most steady and equable supply of moisture and carbonic acid are the most favorable to the growth of plants; and that it is often desirable to moderate the decomposition of rich animal manures by the addition of charcoal, the too rapid disengagement of carbonic acid having a tendency to gorge the plant. If vegetables were really nourished by the direct assimilation of matter previously organized, we should expect that substances which correspond most nearly to the materials of these tissues, such as gum or sugar in solution, would be most favorable to their growth, which is not the case. We are not aware of any instances in which the decomposition of organic matter taken up by the roots has been proved to be performed within the plant; on the contrary, it has been shown that colouring particles are deposited unchanged in the tissues, that the odour of essential oils imbibed by the spongioles is diffused through the whole plant, and that vegetable poisons also traverse the stem and produce their peculiar effects on the irritable organs above, without undergoing decomposition. In all cases, therefore, in which it can be proved that organic matter is absorbed, it can also be shown that it is of such a nature as not to contribute to the nutrition of the plant. It has also been found that the ascending sap of all plants, when examined sufficiently low in the trunk to avoid the fallacy occasioned by the

* Supplement to Alison's Physiology, p. 40.

dissolution of secretions previously deposited in its course, is very uniform in its composition; and this would hardly be the case if it were dependent upon a constantly varying supply of organic matter in different stages of decomposition, such as is afforded by different soils. The opinion that carbonic acid and water form the essential food of plants, is confirmed by the fact that many even of the more highly organized species will grow in circumstances where no other kind of nutriment is accessible to them; and no one is ignorant that the simpler forms of lichens will appear on barren rocks in the midst of the ocean, increasing by absorption from the atmosphere alone, and preparing by their decomposition a nidus for the reception of the germs of higher orders of vegetables. Of the various mineral substances which are taken up in solution by the roots of plants, a proper supply appears to be in many cases essential to the health of the individual; but as they undergo no assimilation or change of composition, and are simply deposited in the tissues, they can hardly be considered as contributing directly to its nutrition.

Some naturalists have contended that all animals do not necessarily derive their support from matter previously organized; but, we must confess that the instances which they have produced in favour of this opinion are far from being satisfactory. It is true that the spatangus fills its stomach with sand, but it derives its nutriment from the minute animals contained in it; the earthworm and some kinds of beetles are known to swallow earth, but it is only to obtain from it the remains of organized matter which are mixed with it; and, in fact, the inorganic matter thus taken into the stomachs of these animals, no more contributes to their nutrition than the gravel swallowed by graminivorous birds, or the chalk eaten by a hen preparing to lay. We are therefore inclined to agree with those who consider plants as intermediate in their constitution between animals and inorganic matter. "The only final cause," says Dr. Roget, "which we can assign for the series of phenomena constituting the nutritive functions of vegetables, is the formation of certain organic products calculated to supply sustenance to a higher order of beings. The animal kingdom is altogether dependent for its support, and even existence, on the vegetable world. Plants appear formed to bring together a certain number of elements derived from the mineral kingdom, in order to subject them to the operations of vital chemistry; a power too subtle for human science to detect, or for human art to imitate."

In our observations on the nutrition of plants, we have hitherto referred to those only which are possessed of a complete system of absorbing and assimilating organs; grafts and parasitic plants form a separate object of consideration. It is well known that it is essential to the success of the process of grafting, that there should be a close alliance in species between the two plants to be united; and hence it is inferred that the ascending sap varies in composition in different families. This may be partly true, since it is known that in rising through the stem it dissolves a portion of the peculiar secretions stored up from the former year; but so many concurrent circumstances influence the result of the operation, that it is difficult to say how much is to be attributed to each. Thus, it is essential to success that there should be an analogy between the modes of growth in the stock and graft; that the two individuals should be naturally in sap at the same period; and that the processes of vegetation should be carried on in both with the same vigour.

The manner in which different species of parasitic plants derive their nutriment, and the analogies which may be traced between them and certain parts of the animal kingdom, would afford a very interesting subject for investigation, a few points of which we shall briefly indicate. "If we examine the influence of living vegetables on one another," observes De Candolle, "we shall see them almost always in a state of contention, less violent and apparent than that of men or animals amongst themselves, but of long duration, and very important in its results." This war is maintained by parasitic plants which live, like carnivorous animals, at the expense of their fellows; though their injurious effect is not always incompatible with the life of the individual upon which they prey.

It is important to draw an accurate distinction between *true* and *false* parasites. The latter vegetate habitually on the surface of other plants, without deriving from them any nutriment except a small quantity of superficial moisture; they will live almost indiscriminately on different trees, or in any situations which will afford them the means of attachment and a small supply of humidity; and, being furnished with their own organs of absorption from the atmosphere, they send no roots into the interior of the plants on which they live. They are therefore only hurtful by covering a portion of the general surface of the tree, by partially shading it from the light if their vegetation is very luxuriant, by affording a nidus to injurious insects, and, in the case of many creeping plants, by acting as ligatures round stems and branches whose diameter is on the increase. Amongst these we may reckon the lichens and other superficial parasites among the cellulares; as well as the ivy and many of the viteaceæ, a large proportion of the tropical orchideæ, and, in general, all plants which have creeping scandent stems.

True parasites, on the other hand, are not furnished with perfect roots of their own, and are therefore obliged to draw more or less of their nourishment from the juices of other vegetables. They may be divided into two great physiological classes; the plants belonging to the first possess leaves or other green parts exposed to the influence of light, and have the power of elaborating for themselves the crude sap derived from the ascending current of the trees on which they grow; the second class comprehends those which are deficient in digesting as well as absorbing organs, and are therefore compelled to derive their food from the juices of plants which have already undergone some elaboration. Of the first class, which may be termed *chlorophyllous* parasites, a characteristic example is the mistletoe, which vegetates on almost all exogenous trees, implanting itself on the bark and sending its radical fibres to the interior of the stem, penetrating and uniting themselves firmly to the woody fibre, with which they form so intimate a connexion that coloured fluids will pass from the stock to this natural graft—for thus it may in some respects be considered. It does not appear that any connexion exists between the missletoe and the bark beneath it, which indeed is always found to be in a state of sphacelation around its insertion. All the parasitic plants of this class (principally belonging to the order Loranthaceæ,) appear to grow with nearly equal facility on a great variety of trees, a fact which we might anticipate from the great similarity which exists between the crude sap of various kinds; but it is remarkable that the mistletoe is very rarely seen to grow on the oak, and it is perhaps from this circumstance that the occurrence was formerly regarded in a religious light.

The greater part of the second or *Aphyllous* class of parasites grow upon the roots or underground stems of other plants, no part of them appearing above the surface (in most cases at least,) except the flower stalks which are occasionally sent up. Amongst this class are the orobanche or broom-rape, and the Lathræa squamaria, whose curious structure has been minutely described by Mr. Bowman.* As they derive their nutriment from the previously elaborated juices of other plants, by suckers fixed in the bark, they are, as we might expect, more restricted as to the number of species upon which they grow.

It appears to us that sufficient attention has not been paid to the physiology of nutrition in forming a natural classification of the vegetable kingdom. If it be true, that plants in general have the power of deriving their nutrition from inorganic matter, and thus of converting into organized products substances much lower in the natural scale, we may conceive them to hold a corresponding rank with herbivorous animals, which, by means of their complex digestive apparatus, assimilate and convert into animal proximate principles, matter previously much inferior in organization. True parasitic plants, on the other hand, like the carnivora, derive from beings as highly organized as themselves, those supplies of nutriment which their simpler conformation requires to have been already partially prepared by other means. The omnivorous animals, too, find a parallel in some of those parasitic species which derive their nutriment partly by suckers applied to other plants, and partly by means of roots sent into the surrounding earth; and the parasitic fungi, like many of the lower kinds both of carnivorous and herbivorous animals, derive their support only from dead or decaying organized substances, and may not unaptly be termed, like insects, "the scavengers of nature."

Plants, like animals, are infested by the growth of parasites within them; and it is a question not yet decided whether the development of these entophytes, which all belong to the fungi, is to be considered as the result of a diseased action in the plant itself, or of the introduction of the germs from without. It has been concluded by Unger,† as the result of his observations on the changes produced by them from their very commencement, as well as of *a priori* argument, that the uredo, œcidium, puccenia, &c. which constitute the appearances known by the names of blight, mildew, smut, &c., are to be considered as the exanthemata of plants, being essentially *maladies des stomates;* and he thinks it questionable whether such productions as the secale cornutum may not be owing to a similar cause. He observes that these morbid growths are most liable to occur in those portions of plants where vegetation is most active, such as the green parts in general, and the leaves in particular; and he remarks that, on the surface of *healthy* bark, we generally find either more perfect cryptogamia, or phanerogamous parasites. The cellular parasites evidently flourish best when the bark is approaching decay; and it often

* Linnæan Transactions, Vol. xvi.

† Annales des Sciences Naturelles. N. S., vol. ii.

happens, that, whilst the stem and principal branches of an old tree are covered by mosses and lichens, these are seen to diminish and disappear as we advance towards the younger and fresher portions. The presence of entophytes is said by Unger to be connected with that of the stomata; and he supposes it to be from some obstruction to their functions that the exanthemata arise. They generally appear at the season of most active vegetation, namely, the spring and early summer; whilst the period for the most abundant and rapid development of the true fungi is the autumn and the commencement of winter. It is but fair to state, however, that Mr. Berkeley, whose authority on such matters is high, considers Unger's statements to be inconclusive.* In those cases in which fungi most unequivocally present themselves, it may be doubted whether they are not rather the consequence than the cause of the disease; for we know that the habit of the class is to appear on matter in a state of incipient or of advanced decomposition; and it would not seem improbable, that like some of the parasites which peculiarly infest the animal body when in a state of weakness, the fungi meet with a nidus fitted for their development in those parts of the vegetable tissue which are undergoing alterations by disease. It is believed by M. De Candolle, that the germs of the entophytic fungi are taken up by the roots and carried along with the current of sap, being deposited and developed in the parts where vegetation is most active; and various facts may be adduced in support of this opinion. We cannot at present dilate more upon this very interesting subject: its close connexion with various doubtful points in animal pathology need scarcely be indicated; and we would suggest it as an important question in *general physiology*, whether plants or animals of a high degree of organization are capable of producing from various parts of their tissues, beings corresponding to those of the inferior orders of their respective kingdoms.†

Though there is no motion of fluids in plants exactly analogous to the general circulation of the higher animals, an investigation into the mode in which the sap absorbed by the roots is propagated to the leaves without a manifest organ of impulsion, cannot but be interesting to the animal physiologist; since it affords him some means of distinguishing the effects of the heart's action from those of other causes which influence the motion of the blood. Philosophers in all ages have attempted to explain the ascent of the sap in plants, some attributing it to simple mechanical powers, some regarding it as an immediate effect of vital agency; and others, with more probability, considering it as partly dependent upon each class of causes. In watching the gradual specialization of the organs of nutrition in the lower orders of cellular plants, we may observe that, as soon as any tendency to vertical elevation is manifested, it becomes necessary that some means should be provided for the transmission of fluid in a determinate direction; and this is accomplished by means of elongated cells, such as we find in the stems of some fungi. The conveyance of fluid thus performed would seem to differ little, except in its possessing a determinate direction, from that which takes place in the cellular tissue of all plants, its hygroscopic properties causing each cell

* Hooker's British Flora. Vol. II. Part II. p. 360.

† See Fletcher's Rudiments of Physiology. Part II. p. 13.

to communicate to its neighbours any superabundance of fluid which it may possess; and it is observable that when the fluid has traversed the elongated cells and arrives at a part of the plant where the vesicles have a rounded form, it is equally diffused throughout. The ascent of the sap in vascular plants appears to take place partly through the young wood, being conveyed from one tubular fibre to another by the hygroscopic, or, to use Dutrochet's expression, the *endosmometric* properties of the tissue; and partly through ducts specially organized for the purpose. These are sometimes of considerable diameter and uninterrupted by transverse partitions, especially in plants having long slender stems, and whose active vegetation requires a rapid transmission of fluid from the roots; the bamboo cane and the vine afford good examples of this structure. It becomes an interesting question to determine by what power fluid is propelled through these vessels, the nature of their parietes forbidding us to suppose that any contraction on their part can be instrumental to the effect, and no evident organ of propulsion existing at their commencement. Our first object, therefore, is to enquire into the causes which modify the quantity of fluid absorbed, and the velocity of its transmission; and it cannot be questioned that the principal of these is the demand occasioned by the vital processes performed in the leaves. Of the fluid conveyed to these organs, the greater part is lost by transpiration, a function partly dependent upon simple evaporation under the influence of heat and a dry atmosphere, and partly upon exhalation from the stomata under the stimulus of light. It has been ascertained by many experiments that the quantity of fluid absorbed by the roots is in strict relation with that transpired from the leaves; and that during the night, and in winter, when the transpiration is wholly at a stand, the process of absorption is almost entirely checked. Even in winter, however, absorption may be artificially excited by any circumstance which occasions a demand for fluid; thus, if a branch of a vine growing in the open air be introduced into a hot-house, its buds being called into action by the increased temperature immediately attract fluid from beneath them, and thus the whole system is put in motion; and De Candolle has shown that in such a case the fluid consumed by the young leaves is really attracted from the cold earth, and not absorbed from the atmosphere of the hot-house. The ascent of the sap in spring may be explained upon the same principle, and we shall quote Dr. Lindley's account of it, which is partly derived from that of Du Petit Thouars.

"In the spring, as soon as vegetation commences, the extremities of the branches and the buds begin to swell: the instant this happens, a certain quantity of sap is attracted out of the circumjacent tissue for the supply of those buds; the tissue which is thus emptied of its sap is filled instantly by that beneath or about it; this is in turn replenished by the next; and thus the whole mass of fluid is set in motion from the extremities of the branches down to the roots. Du Petit Thouars is, therefore, of opinion, that the expansion of the leaves is not the effect of the motion of the sap, but on the contrary is the cause of it; and that the sap begins to move at the extremities of the branches before it stirs at the roots. That this is really the fact, is well known to foresters and all persons accustomed to the felling or examination of timber in the spring; and to gardeners who are occupied with forcing the branches of plants in winter, while their trunks are exposed to the weather." (*Introduction to Botany*, p. 331.)

There is no need therefore of having recourse to the supposition of

*accumulated irritability* for the explanation of these phenomena, which can be so readily accounted for on known principles; and it is obvious that the facts just quoted afford a strong confirmation of the opinion now entertained by many eminent physiologists, that the motion of the blood in the capillaries is very greatly dependent on the changes which it undergoes in them. With regard to the degree in which the motion of the sap is dependent upon the propelling power of the spongioles, we feel much disposed to agree with the views expressed by Professor Henslow:

"The water imbibed by the spongioles is also propelled forward by them with considerable force, and the effects are strikingly analogous to those exhibited by the endosmometer. These effects manifestly bespeak an action very different from the ordinary results of capillarity, and indicate the presence of a powerful force, a 'vis a tergo,' residing in the lowest extremities of the roots by which the propulsion of the sap is regulated. Although these results so closely resemble those of endosmose, there still exists a difficulty in connecting the two phenomena; for, whilst we may admit the possibility of an interchange between the contents of the vesicles composing the spongioles, and the water in the soil which surrounds them, by the ordinary operation of endosmose, it is difficult to explain how the sap may be propelled forward so violently as it appears to be, in the open channels through the centre of the stem, which contain crude sap of nearly the same specific gravity as water itself. It would be further necessary to account for the manner in which a continued supply of fresh materials is obtained for carrying on the endosmose, which must otherwise soon cease when the fluid within has become much diluted. We shall find, however, that a constant supply of fresh material is actually provided by the direct action of the vital force, during a subsequent period in the function of nutrition; and hence it is not impossible, though it has not been proved, that both the propulsion as well as the absorption of the sap may principally if not entirely be owing to the operation of mechanical causes; dependent, however, for their lengthened continuance upon the existence of the vital energy by which those conditions are perpetually renewed, and without which the endosmose would of necessity soon cease." (*Principles*, p. 182.)

The sap in its progress upwards is known to acquire an admixture of matter previously assimilated, either by coming in contact with the descending current, or as Mr. Knight's experiments make it appear, by dissolving secretions laid up in the wood. This admixture may serve the purpose alluded to by Professor Henslow, of increasing the specific gravity of the fluid, and thus maintaining the activity of the process of endosmose; but it also appears to be the first step in the assimilation of the crude materials of the sap. "This assimilation," says Dr. Alison,* "by means of a previously existing product of vital action, is a general law of the maintenance both of animals and plants."

The chemical changes which the sap undergoes in the leaves, by exposure to the atmosphere, have been usually attributed to the function of *respiration*; and as it is now beyond doubt that they are of an opposite nature to those produced by the respiration of animals, many naturalists, and amongst them Cuvier, have proposed to establish upon this contrariety an essential distinction between the two kingdoms. We shall presently show, however, that this opinion may be reasonably doubted. With regard to the aggregate effects produced on the atmosphere by the growth of plants, we may quote from Dr. Lindley an account of Dr. Daubeny's recent experiments, which give a result entirely opposed to those of Ellis.

* Supplement, p. 5.

"Professor Daubeny has ascertained by experiments partially communicated to the British Association, but not yet published, that plants undoubtedly exercise a purifying influence on the atmosphere. In a letter I have recently received from him, he expresses himself thus: 'As the observations of Ellis left it in some doubt whether the balance was in favour of the purifying or the deteriorating influence upon the air, which is exercised by plants during different portions of the day and night, I conducted my experiments in such a manner that a plant might be inclosed in a jar for several successive days and nights, whilst the quality of the air was examined at least two or three times a day, and fresh carbonic acid admitted as required. A register being kept of the proportion of oxygen each time the air was examined, as well as of the quantity of carbonic acid introduced, it was invariably found that, so long as the plant continued healthy, the oxygen went on *increasing*, the diminution by night being more than counterbalanced by the gain during the day. This continued until signs of unhealthiness appeared in the confined plant, when, of course, the oxygen began to decrease. In a perfectly healthy and natural state it is probable that the purifying influence of a plant is much greater; for, when I introduced successively different plants into the same air, at intervals of only a few hours, the amount of oxygen was much more rapidly increased, in one instance to more than forty per cent. of the whole instead of twenty, as in the air we breathe." (*Introduction*, p. 317.)

It has been ascertained from other experiments that a small quantity of carbonic acid is *perpetually* evolved by the leaves both day and night; and the consideration of these phenomena led Professor Burnett to the opinion that, under the name of respiration, two distinct phenomena are confounded. He observes* that the production of carbonic acid is constant during the life of a plant, that it takes place both by day and night, in sunshine and in shade; and that this process, which he considers strictly analogous to the *respiration* of animals, is essential to its existence; for, if deprived of oxygen and confined in carbonic acid gas, vegetables quickly die. On the other hand, the green parts of vegetables, at certain times, and under certain circumstances, decompose carbonic acid, and renovate the air by the restoration of its oxygen; but this occasional renovation may be referred not to the respiratory but to the *digestive* system; and so completely is the process dependent upon the stimulus of sun-light, that plants made to grow in the dark, although they increase in size, do not augment the absolute volume or weight of their solid contents, and even lose some of their carbon by respiration. Here again the analogy holds good between the functions of respiration and digestion in plants and animals; for to both is carbonic acid deleterious when breathed; and in both is it invigorating to the digestive system when absorbed as food.

Plants when exposed to strong sun-light, will thrive in an atmosphere containing 7 or 8 per cent. of carbonic acid; but when placed in the shade they quickly die; and this fact has an interesting connexion with the luxuriance of the fossil flora of the coal formations, which abounds in gigantic specimens of plants whose development is now much inferior, even in tropical climates. It is the opinion of Adolphe Brongniart, that the atmosphere of that epoch was highly charged with carbonic acid, as well as with humidity; and that these vegetables, by the assistance of powerful sun-light, drew from it that nutriment which there was not sufficient soil to afford them, and, by thus purifying the atmosphere, prepared the earth for the residence of the higher classes of animals.

* Journal of the Royal Institution. New Series, Vol. I.

The chemical changes which are produced by the action of the atmosphere upon the crude sap, probably take place in part through the medium of the cuticle of the leaves and other green surfaces; but some are of opinion, that a more direct contact is effected by the entrance of the air through the stomates into the intercellular spaces. Besides this, it is not improbable that the office of the true spiral vessels which are situated in the interior of the stem, is in some way connected with the function of respiration; for the researches of Bischoff have shown that the air contained in them consists of a large proportion of oxygen, which probably discharges some important duty in the economy of the plant; and these vessels are brought into relation with the atmosphere by their communication with the leaves. Hence the respiration of plants appears to bear a close analogy with that of insects, an analogy which was long ago suggested by the spiral structure of the *trachea* in each; but it must be acknowledged that further researches are wanting before we can speak with confidence on the subject.

The animal physiologist will naturally be disposed to enquire whether any development of heat takes place during the respiration of plants; and we might look in vain for any such manifestation during the ordinary performance of the process. There are two periods, however, during the life of a plant, in which respiration goes on with remarkable activity, and in both a considerable degree of heat is generated. In germination, the first of these periods, a large quantity of the surrounding oxygen is converted into carbonic acid, by the liberation of carbon from the seed; and this appears to be the effect of the conversion of fecula or its modifications into sugar, a principle more fit for the support of the young plant. In the second period, that of flowering, the changes which take place are very similar to those produced by germination. A large quantity of oxygen is converted into carbonic acid by the action of the flower; a considerable quantity of heat is developed; and it is believed that the fecula previously contained in the disk is changed by this process into saccharine matter, adapted for the nutrition of the pollen and young ovula; the superfluous portion flowing off in the form of honey. In studying the changes produced during the flowering of plants, we find a direct relation between the carbonization of the air and disengagement of caloric, and the development of the glandular disk. This is especially remarkable in the arum, when a thermometer placed in the midst of a mass of inflorescence has been seen to rise to 121°, while the temperature of the external air was only 66°. From the experiments of Saussure, it appears that the disengagement of heat and the disappearance of oxygen are principally caused by the action of the organs of fecundation; since, when the floral envelopes are removed, the quantity of oxygen consumed by the remaining parts is much increased in proportion to their volume. In one instance, the sexual apparatus of the arum italicum consumed in twenty-four hours 132 times its bulk of oxygen. Unless we admit the existence of a nervous system in vegetables, we can hardly consider the development of heat during germination and flowering in any other light than as a strictly chemical effect arising from the combination of carbon and oxygen; and a strong analogical argument might hence be drawn in favour of the theory which attributes the development of *animal heat* principally to the chemical changes produced by respiration. It has been asked why a disengagement of caloric does not take place in all flowers, if it be produced in this manner; but it should be remembered that in by far the greater number, the heat is carried off by the atmosphere the instant that it is developed; and that it is only where flowers are collected in great numbers within cases which act as non-conductors and confine the heat, as happens in arums, that the elevation of temperature becomes appreciable. M. Raspail, however, offers an explanation of the phenomenon, which differs considerably from that which we have given. He supposes that the elevation of temperature indicated by the thermometer is due, not to caloric liberated from the flower itself, but to the concentration on the bulb of the rays reflected from the interior of the spadix. He supports his view by a series of observations which he made on the comparative heights of two thermometers, of which one was placed in front of a cone of silk, and the other exposed without any envelope; and he states that when influenced by the direct rays of the sun, the former stood nearly 20° above the other. Although we are far from asserting that no allowance is to be made for the source of heat which he has pointed out, we do not regard his experiments as conclusive in favour of its being the only one; since he has neglected the fact that in Hubert's experiments in the Isle of France on the arum cordifolium, the greatest elevation of temperature was always observed at a time when the rays of the sun are least powerful, namely at sunrise; and also that in the experiments of Brongniart upon colocasia odora, the evolution of heat continued to increase rapidly from the complete opening of the flowers to the period of the emission of the pollen.

It appears that the removal of carbon by its combination with the oxygen of the atmosphere is essential also to the ripening of fruit; for when placed in an atmosphere deprived of oxygen, this function is suspended; and if the fruit remains attached to the tree, it dries up and dies. The extrication of carbon appears due, as in germination and flowering, to the conversion of gum and other proximate principles into sugar.

If the hypothesis of Dr. Prout* with regard to the source of carbon in venous blood be correct, a beautiful analogy exists in the effects of respiration on the economy of animals and vegetables. "Gelatine," he says, "which contains three or four per cent. less of carbon than albumen contains, enters into the structure of every part of the animal frame, and especially of the skin;" and as albumen exists largely in the blood, without any gelatine, he considers that "the conversion of albuminous matter into gelatine is one great source of the carbonic acid of venous blood." Now, gum in the vegetable kingdom may be considered to hold a rank very analogous to that of albumen in the animal system; both are immediately formed from the crude nutriment, and serve as the pabulum of the tissues in general; and both are more universally diffused than any other principle. The conversion of albumen into gelatine, therefore, would seem to be a process very similar to the change of gum, and fecula, which is but a modification of it, into sugar.

The fluid which has thus been elaborated in the leaves, partly by the obvious processes of transpiration and respiration, partly by the hidden agencies of vital chemistry, is carried from the leaves by the under stratum

* Bridgewater Treatise, p. 524.

of veins, and appears to descend through the general cellular system of the stem. In Endogens, therefore, it is probably diffused equally through the whole trunk; whilst in Exogens it is principally conveyed downwards by the bark, and carried into the interior by the medullary rays. The descending sap probably contains, ready formed, not only the nutritious secretions destined for the increase of the tissues, but the greater part of those special secretions whose uses to the individual plant the physiologist is at a loss to explain, although their importance in the general economy of nature is so evident. Many of these are stored up in particular receptacles, without our being able to detect any special apparatus by which they are separated from the circulating fluid; but in some cases a distinct glandular structure is very apparent, and the immediate secretions effected by it are collected in an isolated form. The present state of our knowledge does not allow us to state whether these are merely separated from the proper juice, or are the products of a new combination of its elements; but it should be recollected that many vegetable principles are so nearly allied to each other in composition, though totally differing in external qualities, as to be readily convertible into one another by ordinary chemical processes; and we need not, therefore, be surprised to perceive such changes effected in plants without any very evident apparatus for the purpose. It would seem not unlikely from recent investigations into the electrical state of different parts of plants, that electricity has a more extensive influence in the transformation of organic products in the living system than has generally been supposed; and these might be advantageously contrasted with the observations of Donné on the electrical condition of different tissues in the animal body. The recent more accurate analysis of what have usually been termed the proximate principles of vegetables, has shown that many of them are formed by the union of compound electro-positive bases with oxygen, water, &c.; and that the mode of combination in organic and inorganic bodies is not so essentially distinct as was formerly believed.* From the continuance of such discoveries in organic chemistry, aided by well directed enquiries in vegetable physiology, we might not unreasonably look for important results.

The *nutritious secretions*, sugar, gum, fecula, and lignin, may all be regarded as compounds of carbon and water, and differ very little from one another in the proportions of their elements. Sugar, from its crystalline form and simple constitution, is regarded by Dr. Prout as occupying the intermediate place between organic and inorganic compounds; whilst gum, from the universality of its diffusion through the vegetable kingdom, may be considered the essential pabulum of the tissues of plants. Fecula has been shown by Raspail and others to be nothing but a semi-organized form of gum, adapted to be stored up for the nutrition of young parts, without being liable to be dissolved by the fluid circulating around it. With regard to lignin or woody fibre, it must be considered as a still more highly organized product; and if it be true, as Dr. Lindley (following Du Petit Thouars,) supposes, that it does not originate from the descending sap, but is sent down ready formed from the leaves, we should be disposed to remove it from the class with which it is usually asso-

* Turner's Elements of Chemistry, 5th Edit. p. 782.

ciated. Some, however, regard lignin as the matter deposited within the fibre, not as the substance of the tube itself.

The circulation of the *latex* or nutritious fluid, which has been particularly described by Schulz, seems to approach pretty closely to the capillary circulation of animals. We have already mentioned, that there is a difference of opinion as to the nature of the canals in which it takes place; but there is now no doubt of the fact, that an extremely rapid movement of fluids goes on in these passages, in an uninterrupted current passing from one set of vessels to another. As this circulation was first witnessed and described in plants with milky juices, it was supposed by some to be confined to them; hence De Candolle considers it rather in the light of a peculiar local movement. It has been rendered probable, however, by more extended researches, that it is common to all descriptions of plants, and is therefore to be regarded in the light of a general circulation. The currents are very inconstant in their direction, and sometimes stop and recommence suddenly. The causes which produce the movement are quite unknown; it must be totally independent of the impulsion of the spongioles, which can only be supposed to influence the ascending current; it does not seem due to any contractile power in the vessels; and it will continue for a considerable time in detached fragments of a plant. The action is suddenly checked by cold, and again goes on when the temperature rises; by a strong electric shock also the motion is arrested. Further researches are wanting on this subject; but what is already known would seem to confirm the views of those physiologists who regard the capillary circulation in animals as partly independent of the heart.

The *excretions* of plants by their roots, the nature of which has been so successfully investigated by M. Macaire, seem to correspond in character with their peculiar juices, and may be regarded as merely separated from them, in the same manner as (there is now good reason to believe,) the excretions of animals are separated from their blood.* It is probably to the effects of the excretions of one family upon the roots of another, rather than to any peculiarity in the nature of the aliment required by each, that we are to attribute the influence of particular crops upon the soil in preparing or injuring it for the growth of other vegetables. Thus, plants of the family Legumenosæ, which excrete a large quantity of mucilage, prepare or improve the soil for the gramineæ; whilst the papaveraceæ, which excrete a matter analogous to opium, are notoriously injurious to either. If an extensive series of facts of this kind should be established by experiment, it is obvious that the principles of agriculture might be much improved by their practical application.

It is perhaps in the study of the function of reproduction that vegetable and animal physiology are most capable of being advantageously conjoined. In the simplest forms of plants, composed of aggregated vesicles, each cell may be regarded as a distinct individual, being capable of maintaining an independent existence. Here then the processes of nutrition and generation are united, and nutrition may well be called "a perpetual generation." As we rise in the scale, the aggregated vesicles lose a portion of their individuality, the general structure

‡ Alison's Supplement to Physiology, p. 36.

assumes a more determinate form, and a special generative apparatus begins to be evolved; but, even in the higher forms of the Protophyta, there is often no distinct line of demarcation between the nutritive and reproductive portions of the general surface. In the higher cellular plants, however, a more complete separation takes place; but, though the anatomy of their generative system has been attentively studied, and made the basis of classification, little is known of its physiology. In many cases, besides the *spores*, which are homogeneous masses of cellular substance acting like seeds in reproducing the species, the plant propagates itself by *gemmæ*, which may be considered analogous to the leaf-buds of the Phanerogamia. The minuteness of the spores and the universality of their diffusion will probably account for most of those phenomena attributed by some to equivocal generation; and, for a very able discussion on this subject, we may refer our readers to the Library of Useful Knowledge, page 118. In plants having a distinct sexual system, we observe the two modes of reproduction in their most perfect or specialized form; the one connected directly with nutrition, and the other as it were in opposition to it. Each bud of a tree may be regarded as, in some sort, a distinct individual, partly depending on and partly contributing to the general sytem; but, if removed from it, and placed in circumstances favorable to its growth, it is capable of developing all the organs necessary for its support. Hence, the increase of the size of a plant by addition to the number of its buds may be regarded as involving the functions both of nutrition and reproduction; and this increase is directly dependent on the quantity of aliment which it assimilates. The development of the special organs of reproduction, on the other hand, only takes place when the plant is not so highly supplied with nutriment as to occasion the growth of new buds; but it is most interesting to observe that every one of these organs, however various in external appearance, is produced from the same elements as the leaf, and is capable, under particular circumstances, of reverting to that form. We cannot at present enter fully into the theoretical structure of flowers, which is ably discussed in Dr. Lindley's Introduction to Botany; and we must content ourselves with stating the fact that the leaf may be traced through the successive forms of bract, sepal, petal, and stamen, until we arrive at the pistil, whose carpels are manifestly composed of a leaf with its edges folded together. Each of these organs is capable of changing into any other which is intermediate between itself and the leaf, or of at once assuming the form of leaf; and accordingly we find in monstrous flowers, some of which are permanent varieties, numerous malformations of this kind, which distinctly prove the origin of the different appendages. The rudimentary or metamorphosed leaves which constitute the parts of a flower are subject to the same laws of arrangement as ordinary leaves, the tendency of which is to arrange themselves in a spiral round their axis of growth; but, when the latter is not developed, a verticil is formed, as in the ordinary structure of flowers, which normally consist of four or more such whorls. Hence every flower, with its peduncle and bracteæ, may be regarded as a metamorphosed branch. Even the ovules which are developed at the edges of the capillary leaves, which form the placenta, occasionally assume the form of leaf-buds, and then bear a strong analogy with the buds formed upon the margin of some true leaves, such as those of Bryophyllum.

The mode in which impregnation is effected in vegetables is highly interesting. When the pollen is conveyed to the stigma by the bursting of the anther, the agency of insects, or other means, its outer coat soon ruptures, and long slender tubes are protruded through it, which find their way downwards, through the lax cellular tissue of the stigma and style, to the ovarium. The foramen of the ovule, previously to impregnation, is of considerable size; and it appears probable that in all cases it is presented by the adjustment of the ovule, either to the placenta or to that point of the ovary by which the pollen-tubes afterwards enter, so as to receive the direct fertilizing influence of the pollen granules. M. Raspail, however, thinks proper to deny the statements of this process. which were first made by Brown, Amici, Brongniart, and Ehrenberg, and which have been since verified by many observers. We cannot understand the grounds on which M. Raspail bases his assertion, that the contact of the pollen with the stigma is *all* that is necessary for the fertilization of the ovule; since any one possessed of a good microscope and a little patience may satisfy himself of the passage of the pollen-tubes down the style in the Asclepiadeæ, although he may not be able to trace their entrance into the ovarium. In the Coniferæ, however, when no stigma or style intervenes, the pollen-tubes have been seen to penetrate the bottom of the nucleus, when they evacuate their contents; and the evacuated matter soon forms itself into a bag, which eventually contains the embryo. We think it not improbable that the *early* development of the ova of all the more highly organized beings may be eventually reduced to the same type, as has already been accomplished with regard to those of mammalia and birds. Considering the earliest state of the embryo of animals and vegetables as analogous to the simplest permanent forms of their respective kingdoms, and knowing how nearly the latter approach one another, we should expect to find a corresponding approximation in the former, especially as the life of the fœtus until birth may be regarded as almost purely vegetative. Of the further development of the embryo in plants we have already spoken.

The production of *hybrids* in plants is readily effected by the application of the pollen of one species to the stigma of another nearly allied to it. Almost endless varieties may be thus produced; but mules are seldom fertile, except with one of their parents. Many plants, which have been described as distinct species, are either accidental varieties or mules between two varieties; and this fact leads us to doubt whether mules, fertile of themselves, are ever produced between two really distinct species. We are aware that the gardener would bring forward many instances to the contrary; but it must be recollected that a very large number of the plants described as distinct species have in reality a common origin, and are therefore to be regarded as belonging to the same *natural species*, though artifically divided. The tendency of some plants to produce varieties is so great, that many South American specimens, whose history was not known, have been described as distinct species, until it was found that their seeds produced each other indiscriminately. When two permanent varieties differ strongly in form or colour, so as to be regarded as distinct species, which is frequently the case, an immense variety of fertile mules may be produced between them; and it is by such an intermixture of breeds that the races of edible fruits and vegetables have been brought to so high a degree of perfection. If any instance of fertility in true hybrids ever occurs, (of which some instances are on record in animals,) the power appears to become extinct in a few generations.

We intended to dwell at some length on the elementary vital properties of the tissues of plants, with the view of investigating how far they may be regarded as analogous to those of animals: our limits, however, allow us to say but little on this subject. All parts of living plants may be regarded as possessing the property of *excitability*, or irritability, in its most extended sense, exhibiting the various manifestations of life under the influence of external stimuli, and being totally dependent on them for their continuance. In this point of view, they afford to the physiologist the means of tracing the effects of vital stimuli upon organization, more immediately than he can observe them in animals, since there is no good reason to believe that the results are complicated in plants by the influence of a nervous system. The spontaneous motions which occur in plants are generally to be attributed simply to the elastic and hygroscopic properties of their tissues; but the sudden contractions which take place in many instances from the direct application of a mechanical stimulus, would seem to indicate the existence of a property not altogether unlike the irritability of muscular fibre, but insusceptible of any other kind of stimulus.

It is a very interesting subject of enquiry how far the instinctive actions of animals may be regarded as analogous to those of plants. In the systems of the latter, we may observe the direct "respondence of their organization to external agencies;" and the same would be true of the organic functions of animals, if we adopt the opinion of many physiologists, that they are not immediately dependent upon innervation. The powers which Bichat termed (perhaps somewhat incorrectly) organic sensibility, and insensible organic contractility, may thus be considered as the proximate causes of the manifestation of *vital instincts* both in plants and animals, whatever we may think of their relation in the latter with the ganglionic system of nerves; and we might then regard the obvious motions of plants as occasioned by the exaltation of these powers in certain cases; whilst the *external instincts* of animals, requiring for their manifestation the exercise of the organs of voluntary motion, are obviously dependent upon the nervous system, by which alone these organs can be made to act. But, as Mr. Kirby justly remarks, "can we not conceive that the organization of the nervous system may be so varied and formed by the Creator as to respond in the way that he wills to the impulses of the physical powers of nature, so as to excite animals to certain operations for which they were evidently constructed?" The subject upon which we have thus cursorily dwelt is one which offers a wide field for investigation; and we hope that we have succeeded in showing that such an enquiry may be conducted most philosophically, and with the greatest prospect of success, when it is made to embrace all the classes of objects in which vital phenomena are manifested.

